# Memory, modelling and Marr: a commentary on Marr (1971) ‘Simple memory: a theory of archicortex’

**DOI:** 10.1098/rstb.2014.0383

**Published:** 2015-04-19

**Authors:** D. J. Willshaw, P. Dayan, R. G. M. Morris

**Affiliations:** 1 School of Informatics, University of Edinburgh, Edinburgh EH8 9LE, UK; 2 Gatsby Computational Neuroscience Unit, University College London, London WC1N 3AR, UK; 3 Centre for Cognitive and Neural Systems, University of Edinburgh, Edinburgh EH8 9JZ, UK

**Keywords:** hippocampus, theoretical neuroscience, memory

## Abstract

David Marr's theory of the archicortex, a brain structure now more commonly known as the hippocampus and hippocampal formation, is an epochal contribution to theoretical neuroscience. Addressing the problem of how information about 10 000 events could be stored in the archicortex during the day so that they can be retrieved using partial information and then transferred to the neocortex overnight, the paper presages a whole wealth of later empirical and theoretical work, proving impressively prescient. Despite this impending success, Marr later apparently grew dissatisfied with this style of modelling, but he went on to make seminal suggestions that continue to resonate loudly throughout the field of theoretical neuroscience. We describe Marr's theory of the archicortex and his theory of theories, setting them into their original and a contemporary context, and assessing their impact. This commentary was written to celebrate the 350th anniversary of the journal *Philosophical Transactions of the Royal Society*.

## 1. Introduction

**Figure 1.**
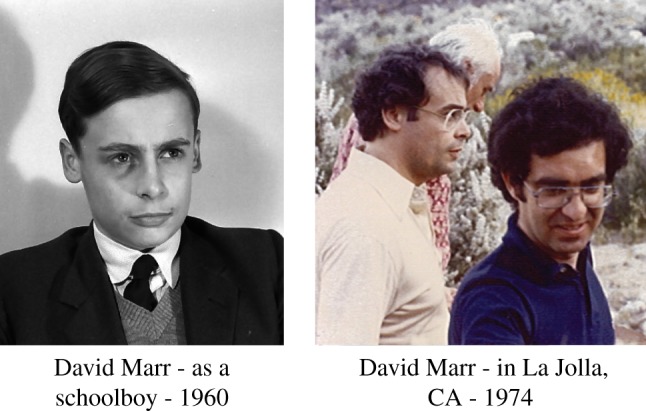
Photographs of David Marr. (*a*) At school, aged about 12. (*b*) David Marr (left) with his colleagues Francis Crick (back) and Tommy Poggio in California, 1974. Reproduced with kind permissions of Peter Williams (*a*) and Lucia Vaina (*b*).

After burning so brightly with his neuroscience work and his later contributions at the birth of the field of computational vision, David Marr (figure 1) died tragically young. His archicortex paper comes from the initial phase of his career, in which he constructed whole intellectual edifices concerning how the cerebellar cortex, the neocortex and the hippocampus could function as networks for learning and memory. In his first paper, he discussed how the Purkinje cells of the cerebellar cortex could learn motor commands [1]. He then outlined how the neocortex could perform unsupervised learning to store highly processed information about inputs and thereby extract categories [2]. His third theory described how the archicortex, the hippocampus and associated structures, could function as a *simple memory* by storing information directly and temporarily, prior to further processing in the neocortex [3]. It was this paper that was published in the *Philosophical Transactions*. A posthumous book contains a notable and comprehensive collection of these papers and his other work, including invited commentaries [4].

The three early papers were strikingly different from all that had come before, and indeed most since: Marr articulated computational problems, posed in mathematical terms, to be solved by these structures; he suggested how existing anatomical and physiological knowledge related directly to the computations; finally, he nailed his modelling colours firmly to the mast, making numerous predictions that were starkly graded according to the severity of their consequences for the theory were they to be refuted.

Have such refutations indeed happened? We examine this question with a focus on his theory of the hippocampus [3], also looking more widely at how it has stood the test of time. Despite being wrong in some details, this work has been hugely inspirational for other theories and experiments. Marr's early studies are of even greater note for the concerns about the overall philosophy and practice of *modelling* that were inherent in the original three papers, and that he and Poggio, his close colleague, later crystallized [5]. This analysis was reported in the book *Vision* [6]. Their ideas about different levels of analysis—*computational, algorithmic* and *implementational*—have much resonance today, and are especially relevant to the current debate within the neuroscience community about attempts to build large-scale models of the brain.

Already in his schooldays, David Marr was developing his interests far beyond those provided by his formal education in mathematics and physics. Stimulated by reading books such as J. W. Dunne's *An Experiment with Time* and W. Grey Walter's *The Living Brain*, he became enthused about the possibility of a ‘mathematical theory of the brain itself’. He then went up to Trinity College Cambridge as a mathematics scholar. After graduating, he studied physiology and anatomy before becoming a PhD student with Giles Brindley who inspired him to produce his first theory paper on the cerebellar cortex. He then secured a Prize Fellowship at Trinity; notably, three of the four awards that year were to neuroscience, the other recipients being Tony Gardner-Medwin and Oliver Braddick—later to become a distinguished physiologist and experimental psychologist, respectively. During this Fellowship, he completed his two other early neural papers including the theory of archicortex. In 1970, he joined Sydney Brenner at the MRC Laboratory for Molecular Biology at Cambridge. Sydney encouraged him to experience at first hand the rising discipline of Artificial Intelligence by visiting Marvin Minsky and Seymour Papert at MIT. This eventually led him to shift his focus towards computation rather than implementation, and to his long partnership with Tommy Poggio. Following a brave battle with leukaemia, David Marr died in 1980.

### 2. Mathematical and computational modelling in neuroscience

There are three main varieties of theoretical approaches in neuroscience: *data analysis*, *mathematical modelling* and the one that became clearer through Marr's later work, namely *computational modelling*.

*Data analysis* involves developing and deploying advanced computational and statistical methods for analysing the gargantuan volumes of data now being generated by an ever wider variety of experimental techniques and assessing the complex interactions among the multiple entities contained within these data.

*Mathematical modelling* involves building formal reductions based on descriptive and mechanistic models of natural phenomena associated with the brain over the huge range of spatial and temporal scales that characterize it. These reductions have to take into account the complexity and heterogeneity of the brain's components. Mathematical methods and computer simulations are used to explore whether the mechanisms proposed are capable of accounting for the phenomena they are intended to explain.

*Computational modelling* most interested Marr. He looked upon the brain as a physical device that is performing computational tasks involving representing, processing and acting upon information. That the brain can be interpreted as processing information provides a rich set of constraints on the mathematical models, whose structure and dynamics have to be appropriate to accomplish the computational tasks.

Marr distinguished three levels of computational modelling—implicitly in his early writing, but later, transparently. Examples are given in table 1:

(1) The *computational level*, at which the task and the logic of its solution is described;
(2) the *algorithmic level*, which specifies how the information associated with the computation is represented and the procedures for performing the relevant manipulations; and
(3) the *implementational level*, which describes how the algorithms are realized in the nervous system.

**Table 1.** Examples of ‘…the different levels at which an information-processing device must be understood…’ [6] from which example 1 was taken. Example 2 is based on Li and Zhaoping [7,8] and example 3 on Daw *et al.* and Montague *et al.* [9,10].

computational	algorithmic	implementational
1. performing addition	using Arabic numerals, adding the least significant digits first; using a binary representation	using a machine with 10-toothed wheels; using logic gates
2. visual salience	assessing where the statistical structure of images changes	dynamical interactions between hypercolumns in V1
3. optimal control	learning a model of the world and planning using the model; learning the future worth of current actions	state-based prediction errors and working-memory for tree search; temporal difference prediction errors realized in the phasic activity of dopamine neurons

One recurring theme in his work was the interaction between levels. This was fluid in his early work, as seen most clearly in his cerebellar cortex theory, which he developed to address the beautiful three-dimensional structure of the neuronal network of the cerebellar cortex [11], and that had such an impact among cerebellar physiologists. Equally, his neocortical theory blended categorization, as an implication of the exciting observations from Hubel & Wiesel of single neurons in the visual cortex responding selectively to moving lines and edges tilted at a particular angle [12], with statistical notions of this operation associated with the then emerging field of numerical taxonomy, an established focus of research at Cambridge [13]. This was all under the guidance of a very careful analysis of neocortical anatomy as implementation. The algorithmic rendition of his theory of the hippocampus was strongly influenced by what was then known about the neuroanatomy, at least up to an impressive point. Some facets were omitted—for instance, it did not incorporate the substantial mechanistic complexities of actual neural elements known at the time, such as the details of spike generation or synaptic integration via extended dendritic trees.

Marr's subsequent work, perhaps influenced by the Artificial Intelligence community at MIT, and perhaps as a reaction to the overly precise predictions made from the neurobiology in these earlier papers, focused on insulating computational and algorithmic levels from the requirements of implementation [6]. Indeed, a tenet of computational theory is that the same algorithm can be implemented in distinct ways in different hardware. Read strictly, this could imply that it is impossible to generate strong constraints that apply across computational levels, a restriction that would undermine the programme of theoretical neuroscience. However, recent thinking has recognized the potential for well-founded computational accounts that exploit both weak and strong constraints across levels, and that furthermore tightly integrate computational and mathematical modelling [14–16].

This discussion of Marr's approach provides a basis for understanding the sort of theory of the hippocampus for which he was aiming, and equally a framework within which to evaluate the extent to which these aims were met. After discussing the model and its impact in these terms, we will return to assess the wider impact of Marr's modelling philosophy and methodology.

## 3. Marr's theory of archicortex

Marr regarded the archicortex's computational task as acting as a temporary store of raw information derived from sensory experience: the hippocampus should memorize patterns of neural activity representing events as they happen through the day, with previously memorized patterns being retrieved when cued by partial information. According to his neocortex theory, the information stored in the hippocampus would then be transferred periodically to the neocortex, to be recoded via self-organization into a more categorical form [2]. While the hippocampal model, therefore, lacked the sophistication of the neocortex, building a model of ‘simple memory’ was nevertheless, he asserted, still a ‘necessary technical exercise’. A more detailed explication of this exercise is provided in Willshaw & Buckingham [17].

As in Marr's two other contemporary neural models, the archicortex model comprised several layers of interconnected neurons with the connections specified probabilistically. This network structure can be traced to the more abstract Perceptron [18], an elegant mathematical account of the latter's capabilities having just been published by Minsky & Papert [19]. The items to be stored in the model (called *events*) are represented by patterns of activity over the so-called *input* layer of neurons, storage being achieved through synaptic plasticity. Retrieval occurs when a portion of a previously stored event is presented to the input layer. Activity is propagated to all the remaining layers through synapses connecting one to the next, ultimately leading to a pattern of excitation over the neurons in the final (or *output*) layer. This output pattern is called the *simple representation* of the input pattern. It is then passed through another set of return synapses of variable strength directly back to the input layer. The synapses each have a binary-valued modifiable component, being strengthened by the coincidence of presynaptic and postsynaptic activity. Together with these Hebbian synapses [20], Marr proposed the existence of synapses with a weak or ‘baseline’ unmodifiable component that would enable postsynaptic cells to fire when activated (Brindley synapses—named after Marr's PhD advisor, Giles Brindley).

On the basis of reasonable calculations, Marr asserted that events could be stored no faster than one per second, and that information would be transferred to neocortex overnight, when there would be no sensory input to provide unwanted synaptic modification. He, therefore, set the memory capacity at 10^5^ (approx. the number of seconds in a day). He estimated the number of neocortical pyramidal nerve cells to be used as the input layer at 10^5^, and the number in the output layer at 10^4^. Arguing that there would be no capacity for the return synapses to take part in pattern completion during retrieval, he assumed that the simple representation was completed in the output layer before being fed back to the input layer. No detail was provided about the return projection—the intended topic of a subsequent paper that sadly did not materialize.

Using his main mathematical result from the cerebellum paper concerning the number of events that can be stored and retrieved by a single Purkinje cell, he calculated that a simple two-layer model with the modifiable synapses connecting the input layer to the output layer directly would be inadequate, as the proportion of nerve cells active (the *activity level*) in a simple representation would be too low to be reliable.

He, therefore, concentrated on a three-layer model, with information flowing from input to output through a middle or *codon* layer. He assumed that there was a recurrent feedback loop with modifiable synapses between output layer neurons. The resulting *collateral effect* enabled a partially reconstructed simple representation to be improved gradually, so that the full simple representation could be reconstructed and sent back to the input layer through the return pathway.

Marr sought to ensure that (i) the activity levels in the various layers were not too low; and (ii) each event had a unique representation in each layer. This implied mathematical constraints on the parameter values of the system—principally the activity levels in each layer, the number of cells in the middle layer and the density of connections within each group of synapses. It then became crucial to set the firing thresholds for each nerve cell receiving input through modifiable synapses, something that he suggested, as in the cerebellum paper, could be achieved by a combination of divisive (somatic) and subtractive (dendritic) inhibition. In the reconstruction of the simple representation in the output layer, some cells may be inactive when they should be active, whereas others may be spuriously active; via what was described candidly as ‘suitable juggling’ of the thresholds [3, p. 80], the number of genuinely active cells would be expected to increase and the number of spuriously active cells to decrease, maintaining a constant activity level, until the full simple representation emerges with an accuracy of a few per cent. Appropriate settings for the thresholds depend on information about both the number and proportion of a nerve cell's afferent synapses that are active in storage or retrieval. Marr calculated possible values of these two parameters that, in combination, would yield the desired memory capacity. He made calculations for different configurations of (probabilistic) connectivity. In some configurations, connections between all cells in one layer were allowed to the cells in the next; in others the connections were restricted, to reflect the topographic organization believed to exist within some parts of the archicortex. All calculations were made for 10^5^ cells in the output layer rather than the figure of 10^4^ used initially when he rejected the two-layer model.

**Figure 2.**
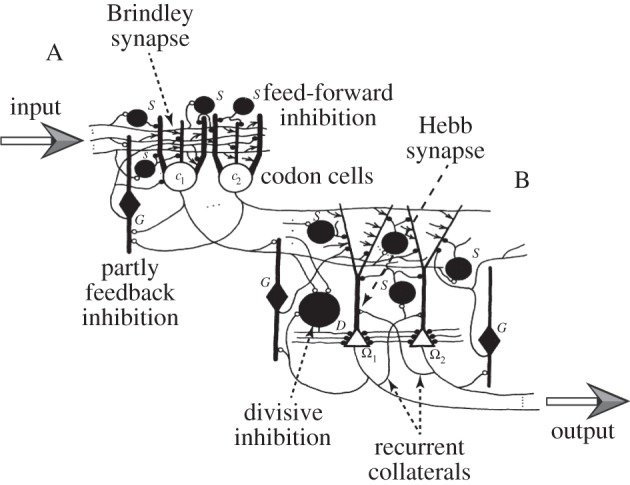
Schematic of a basic unit of Marr's simple memory model. The basic unit has two conceptual, connected parts, input (labelled A) and output (B). ‘A’ shows the horizontally running fibres from the input layer with modifiable Brindley synapses on cells of the intermediate layer (‘codon’ cells, of which two are shown, *c*_1_ and *c*_2_). Inhibitory interneurons control the threshold for codon cell firing so as to maintain a constant activity level. Neurons of type *S* and *G* supply feed-forward inhibition by the sampling of input fibre activity; those of type *G* also provide feedback inhibition by sampling the codon cell activity. Using feedback and feed-forward inhibition for controlling thresholds in this way was used by Marr in his cerebellum paper [1]. ‘B’ shows codon cell fibres with modifiable synapses on output cells Ω_1_ and Ω_2_. Collateral connections from one output cell to another are also indicated. The threshold of firing on the output cells is controlled by *S* and *G* interneurons, as above. In addition, the *D* cells innervate the soma to perform a division. Both subtraction and division are needed for correct threshold setting of the output cells, by means of which the correct simple representation is gradually recreated from a partial input cue. The return projection from output cells to input cells is not shown. Adapted from fig. 5 of Marr [3].

Marr incorporated the then available knowledge of the neuroanatomy of the hippocampus and related brain structure in great detail. In particular, he suggested that it is the anatomy of the principal cell types that determines the function of the archicortex to be a memorizer (in the same way that the different cell types of the neocortex specify it as a classifier). The paper is replete with diagrams showing connectivity among neurons within the hippocampal formation (figure 2). The input layer was proposed to be the pyramidal cells in neocortex, the stellate cells in entorhinal cortex and presubiculum formed the codon layer, and the output layer embraced the dentate gyrus and the CA1, CA2 and CA3 pyramidal cells of the hippocampus; modern interpretations identify the codon layer with the dentate gyrus and the output layer with CA3, where there is a known feedback loop. Marr also suggested how particular memorizing cell types could be used for threshold setting through inhibition. Finally, he furnished a large list of predictions accompanied by numbers of stars: a three-star prediction would dismantle the theory were it to be disproved; a no-star prediction was merely a strong hint.

## 4. Marr's theory in its own time

Marr's theory hails from an era when much less was known about the psychological and computational roles of the hippocampus, and indeed its neurobiology. However, there was still a considerable contemporary psychological and physiological context which he did not mention (perhaps a relief to the reader of what is an intricate paper, lacking the relaxed style of his book *Vision* [6]).

The development of Marr's *simple memory* idea may have been influenced by the striking observations made on a series of patients who had been given bilateral surgical resection of the temporal lobes for the relief of epilepsy [21]. The best known of these, Henry Molaison (patient HM), experienced relief from seizures following the operation but, more strikingly, he displayed profound *anterograde amnesia*: while he could hold information in short-term memory for a few minutes, he could not form long-term memories. Detailed study of HM through the 1960s until his death in 2008 by Brenda Milner, Suzanne Corkin and their students substantiated and elaborated upon the initial clinical observations [22].

This unexpected finding led immediately to attempts to model the syndrome in non-human primates and rats. These efforts were largely unsuccessful as there was no deficit in learning after damage to the hippocampus. Indeed, contemporary hypotheses suggested that the hippocampus may be a behavioural inhibition system [23] on the basis that hippocampal lesioned rats could learn but had great difficulty in either giving up or changing learned habits. It was not until some years later that the first successful animal models of amnesia were developed [24,25], building on the idea that memory for events and the capacity to acquire new habits are mediated by distinct neural systems. Either Marr did not know of these initial unsuccessful attempts to model the syndrome or, wisely ignoring them, he focused on the fascinating anatomy of the hippocampal formation as being ideal for ‘simple memory’—keeping track of the events of the day in precisely the way that patient HM could not.

### (a) Evaluation of the modelling

As noted, Marr's archicortex model bears a strong family resemblance to his three other network models of learning and memory with their three layers of nerve cells (input→codon→output), linked together randomly with modifiable synapses. They use the same mechanisms for synaptic modification and for setting the thresholds on the firing of cells in the codon and output layers. The principal differences between the three lie in whether they act as classifying or memorizing devices (determined by the anatomy) and the use to which the codon layer is put.

In the cerebellar model, the single output cell is taught to respond selectively to many different input patterns. An efficient way of producing high storage capacity is to use patterns with low activity levels. The codon layer acts to transform the input patterns with high but variable activity levels into patterns of more constant and lower activity so as to make any two patterns more distinct from one another (*pattern separation*). This is most easily done if the codon layer contains many more neurons than the input layer, as in the cerebellum. Marr calculated that 7000 mossy fibres (inputs) would influence the single Purkinje cell (output cell) through 200 000 granule cells (codons), achieving a reduction in activity level from between 1 and 25% in the input to 0.25% in the codon layer. By contrast, in the neocortex model, the codon cells act to pick out common features of the input patterns to enable each output cell to learn all patterns of a particular class. For the archicortex model, without ascribing a specific function to the codon layer, he may have thought it would facilitate *pattern completion* (recovery of a stored pattern from a fragment). There is no pattern separation as the activity levels are roughly the same in each layer of the model.

Marr's calculations showed that in his model, simple representations could be established and recalled from partial information. Had it been possible to carry out computer simulations, he might have been able to extend his model to: (i) investigate how to incorporate the missing final step in his model of reconstructing the input pattern through the final feedback pathway and (ii) specify a working threshold-setting strategy. Most significantly, had he kept the same number of output layer cells (10^5^) from the outset, rather than switching from 10^4^ to 10^5^, there would have been no reason to reject the two-layer model. One major simulation which confirmed the validity of his assumptions (*albeit* on a 1/100 size model) found that the performances of two- and the three-layer models were broadly equivalent [17,26]. His choice of a three-layer rather than a two-layer model seems to rest on using constraints from an implementational rather than a computational perspective. Similar simulation techniques verified Marr's calculations for the cerebellum model [27] and explored the computations performed in the neocortex model [28].

Marr discussed how to clear the memory periodically, but without coming to a clear algorithmic solution. Instead of setting all synapses to zero overnight once the patterns had been re-presented to neocortex, he considered either the selected deletion of the synapses activated by particular simple representations or the gradual decay of all synapses (which he said requires simpler assumptions). Subsequent work by a variety of authors has shown that having all synapses decay lowers memory capacity [29–31].

## 5. Marr's theory in modern terms

Later in his career, Marr became focused on the computational level, leading to much debate about his own views about his earlier models. At the very least, we can see this work as a noble failure—it was an astonishing achievement for a mathematician to synthesize so much disparate data into a whole. A later review reminded a new generation of neuroscientists how the combination of specific features of hippocampal anatomy coupled with activity-dependent synaptic plasticity could mediate distinct components of memory [32]. It also discussed further concepts such as pattern completion, pattern separation and the role of sleep in memory reactivation, all mentioned in Marr's original works.

### (a) Systems consolidation

Marr's theory that the archicortex acts as a temporary store to enable events to be appropriately recoded in the neocortex lies firmly within the domain of long-term memory, rather than being a limited capacity short-term memory of the kind used to remember, e.g. telephone numbers. Its interaction with neocortex is now referred to as ‘systems memory consolidation’. This has been the subject of experimental work for over a century, but only more recently considered theoretically [33]. The complementary learning systems framework of McClelland *et al.* [34,35] is perhaps the best worked out computational model of systems memory consolidation, and is very much in the spirit of Marr's original ideas. Consolidation is best seen as a process by which memory traces become stabilized and integrated into neocortical networks that sustain and expand upon the memory. The standard view is that this process is a long and gradual one [33], whereas according to Marr it would happen over the course of a single night. This distinction has quantitative implications for understanding the temporal characteristics of *retrograde amnesia*, which refers to the forgetting of information learned prior to damage to the hippocampus. The experimentally somewhat controversial prediction is that older memories should be remembered more proficiently.

The consolidation idea has been refined in several ways. First, the concept of ‘transfer’ of information from hippocampus to cortex is no longer accepted. Instead, parallel encoding is now considered explicitly, with hippocampal–neocortical interactions serving to stabilize neocortical traces selectively. Second, according to a multiple trace theory, event memory can be subdivided into context-specific episodic memory that is stored in hippocampus (‘what, where and when’), and semantic memory for ‘facts’ stored in neocortex [36]. Episodic memory is analogous to Marr's recording of events—the memory of things that happen during the day. Semantic memory is the corpus of factual knowledge acquired through formal training or from our interpretation and recoding of events. Third, new work on frameworks of semantic knowledge in both animals and humans suggests that activated prior knowledge can guide or at least influence the process of systems memory consolidation, and thereby decrease the time it takes [37,38]. These challenge the notion that the simple memory is the only fast component of the system. Even in the complementary learning systems framework, the gradual creation of categories in neocortex occurs using plasticity mechanisms that are just as fast in the neocortex as in the hippocampus [34].

### (b) Spatial memory

One important algorithmic assumption was that the hippocampus and related structures are incapable of generating systematic representations of their own and instead merely inherit random representations from neocortical input. The first hints that this is incorrect had already been evident in the discovery of hippocampal place cells [39]. This led to the suggestion that the hippocampus processes places and contexts [40]. The observation of place cells was followed by that of head-direction cells reflecting directionality [41,42]; and of grid cells which provide a metric for transitions through space [43,44]. Research on the human brain, using both intracranial electrophysiological recording and functional magnetic resonance imaging (fMRI) has confirmed the presence of place and grid cells in humans [45–47]. The huge interest in spatial learning and memory continues to this day.

This research has led to the conjecture that critical processing by the hippocampal formation includes representation of location within an orientationally anchored metric representation of space, with recovery of past events involving remembering where they took place. Much recent neural circuit analysis is endeavouring to work out implementational details. How discrete events are represented, and how they are anchored in present or future time and space to a specific context, remains poorly understood—though the notion of the hippocampus acting as a type of distributed associative memory [48–50] that binds events to context has been discussed extensively in the neuroscience literature [32,51,52]. Certainly, the recovery of the stored representation of an event may now be seen to be one that involves remembering where that event took place, a less abstract process than that envisaged by Marr.

### (c) Cellular and subcellular processing

Marr provided what looked like a very clear guide to testing the implementation of his model. However, it actually turns out to be very difficult to do so convincingly. For instance, the degree of abstraction necessary in treating all the complexities of the internal connections within the hippocampus as being just part of the output layer renders many of the implementational suggestions rather moot. Thus, it can be criticized even at the coarse level of connectivity at which the model could have made contact with the neural substrate—the more so for lacking any information about the input–output relationship with the neocortex. The theory was on far firmer ground at the level of synaptic plasticity; and indeed, Marr mentioned (in a footnote) Lømo's initial observations about synaptic potentiation later collected and expanded in the well-known paper of Bliss & Lømo [53].

**Figure 3.**
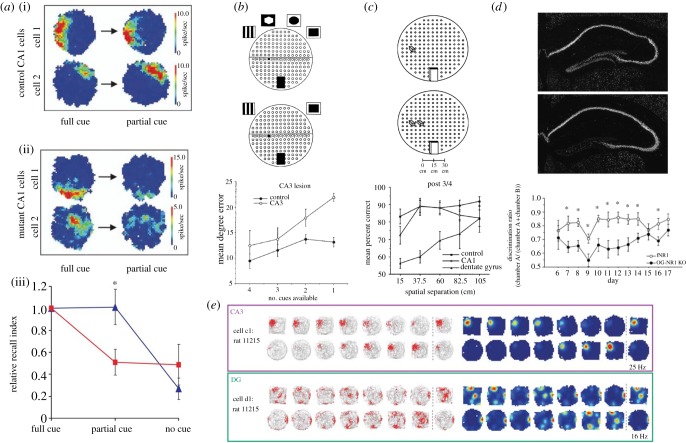
Complementary experimental work on pattern completion and pattern separation using different techniques. (*a*,*b*) Pattern completion. (*a*) Deletion of NMDA receptors in area CA3 leads to problems finding the learned location of a hidden platform in a water maze only when most cues are removed (partial; lower). Place fields (the spatial receptive fields of place cells) in area CA1 lose their integrity in the same circumstances (from Nakazawa *et al.* [71], reprinted with permission from AAAS). (*b*) Experimental apparatus for examining pattern completion for spatial information (top). Rats were shown an object in one location (in the middle row, shown by the little black object) during a ‘sample’ trial and then had to find its location again in a later ‘choice’ trial when some of the external cues were absent. Lesions of area CA3 led to a parametric impairment depending on the number of absent extra-maze cues (from Gold & Kesner [72], used with permission from Wiley-Liss, Inc.). (*c*,*d*) Pattern separation. (*c*) In a similar apparatus to (*b*), rats performed a delayed match to sample task for the location of an object given an identical distractor object at various separations. Lesions of the dentate gyrus caused a distance-specific deficit in this task (bottom) (from Gilbert *et al.* [73], used with permission from Wiley-Liss, Inc.). (*d*) A molecular engineering approach showing selective deletion of the NR1 subunit in the dentate gyrus (top panel is a control mouse, bottom panel shows deletion). These mice displayed a difficulty in discriminating two contexts in a fear-conditioning task (from McHugh *et al.* [74], reprinted with permission from AAAS). (*e*) Changes in the distribution of rates of firing of simultaneously recorded neurons in CA3 (right, top) and DG (right, bottom) in an apparatus that could be gradually changed in shape from a square to a circle. Panel shows trajectories of the animal and firing rate (left) and colour-coded rate maps (right). Note changes in rate of firing of CA3 cell but ‘re-mapping’ by the DG neuron (from Leutgeb *et al.* [75], reprinted with permission from AAAS).

Certainly, he was prescient in imagining that the axonal targets of inhibitory neurons could be on the cell soma or the dendrites (e.g. figs. 3–5 of [3]). It took many years and the elegant work of Somogyi and his colleagues in Oxford before their combined electrophysiological, immunocytochemical and ultrastructural studies at the single-cell level confirmed that distinct types of inhibitory neuron within the hippocampal formation (now at least 25 types) have differential patterns of connectivity [54].

The existence of oscillatory activity in the hippocampus had been established at the time of Marr's paper, but he made no reference to it. A theta rhythm (5–12 Hz) could be gating the memory-encoding activity of N-methyl-D-aspartate (NMDA) receptors [55], or acting as a rapid phase-regulator of encoding and retrieval [56]. Additional noteworthy rhythms include hippocampal sharp waves seen in field-potentials, which Buszáki [57] has suggested may be mediating hippocampal–neocortical interactions during consolidation and which have also been implicated in hippocampal replay after sleep [58]. New work has suggested that gamma rhythms (30–80 Hz) may play a role in gating the inputs to the hippocampal formation from layer II and layer III of the entorhinal formation [59]. All these ideas are at the forefront of experimental work with many details to be worked out, but it seems certain that Marr would have been excited by this more ‘dynamic’ conception of his simple memory.

An implementational absentee from Marr's theory was neuromodulation. As well as their possible role in novelty, dopamine neurons are also known to report prediction errors associated with rewards [60], with similar potential consequences for memory [61,62]. Acetylcholine has been implicated in modern versions of Marr's theories, regulating synaptic drive and efficacy so that retrieval of existing memories and storage of new ones can be appropriately separated [63–65].

Regulation of synaptic efficacy is central to the capacity of the memory to store information. Of the two types of synapse envisaged by Marr, the Hebb synapse [20] was later identified with long-term potentiation (LTP; [53,66]); the Brindley synapse may exist but there is no firm evidence. Since 1971, once the actions of glutamate on the four receptor subtypes, AMPA, NMDA, kainate and mGLUR were understood (GLU-A,N,K and M, respectively), many aspects of synaptic plasticity such as *associativity* and *cooperativity* could be accounted for. Marr might have been intrigued that the NMDA receptor has the biophysical properties necessary for detecting the conjunction of presynaptic activity and postsynaptic depolarization, that is then signalled with a different ion (Ca^2+^) from that mediating fast synaptic transmission by AMPA receptors (Na^+^); and that metabotropic glutamate receptors could inform postsynaptic signalling cascades about the magnitude of presynaptic input without regard to the level of postsynaptic depolarization. In addition, the hippocampal slice preparation, allowing studies in which drugs could be washed out as well as into living brain, led to increased understanding of activity-dependent synaptic plasticity, including the critical role that NMDA receptors play at the time of induction [67]. Synapses are now regarded as bidirectionally modifiable, exhibiting both LTP and long-term depression (LTD), which has been shown to have implications for storage capacity [68]. There are numerous observations such as that blocking NMDA receptors impairs memory formation [69], and the recent finding that synaptic plasticity is also shown by inhibitory interneurons [70].

### (d) Pattern separation and pattern completion

Arising out of the modern interpretation of the dentate gyrus and CA3 cells as the codon layer and the output layer, respectively, there has been both experimental and theoretical work on the role of the collaterals in taking partial patterns and returning their original, complete, matches. One such study involved the restricted genetic ablation of NMDA receptors on pyramidal neurons in CA3 in mice, leaving fast synaptic transmission intact but impairing plasticity [71]. When trained on a spatial task with a rich set of extra-maze cues, the knock-out mice were impaired when required to recall with all but one cue absent (figure 3*a*). In rats whose CA3 had undergone an excitotoxic lesion, there was a parametric disintegration in performance when required to remember a maze location for a short period of time as more spatial cues were removed (figure 3*b*; [72]).

Similar studies have been used to examine pattern separation, a function that Marr studied in great detail for his cerebellum model. The modern notion is that granule cells of the dentate gyrus (DG) have a similar function to granule cells in Marr's model of the cerebellum in mapping similar input patterns to dissimilar representations. One study showed that rats trained to distinguish the locations of two objects were impaired selectively after DG lesions as an inverse function of the spatial distance between the objects, suggesting that pattern separation had been impaired (figure 3*c*) [73]. Likewise, selective deletion of the NR1 subunit of NMDA receptors in the DG of the mouse (impairing plasticity rather than activity) resulted in animals able to learn a context fear-conditioning task, but unable to distinguish two similar contexts associated with the learning task (figure 3*d*) [74]. Single-unit recordings from CA3 (figure 3*e*, upper) and DG (figure 3*e*, lower plot) by Leutgeb *et al.* [75] have shown that signals from the entorhinal cortex could be decorrelated by changes in the pattern of firing in the dentate gyrus by the recruitment of non-overlapping cell assemblies in CA3, consistent with the expectations of Wills *et al.* [76]. Similar studies in humans, using fMRI, have also been conducted [77].

### (e) A broader view of archicortex

Other computations have also been ascribed to the archicortex. For instance, it may be as involved in constructing (i.e. imagining) possible future events as it is in reconstructing (i.e. remembering) past events [78–81]. This implicates it in mechanisms for planning, such as of possible trajectories in space [82,83]. Equally, the hippocampus might offer abstract representations for sensory stimuli [84,85], effectively binding together disparate information about objects in just the way that place cells bind together disparate information to generate a code for location [40]. A third idea is that the hippocampus is a comparator, detecting and highlighting unpredictability and anomalies. This has implications for issues such as anxiety [86], and the influence of novelty on memory processing [55,87]. Dopaminergic neurons in the ventral tegmental area (one of the neuromodulatory absentees mentioned above) might communicate novelty to the hippocampus and thereby enhance the synaptic persistence of associated events as outlined in the synaptic-tagging and capture theory [55,88,89], although the possibility of a contribution by other neuromodulatory afferents needs also to be considered. In either case, the simple memory could hold information for longer periods and enable the overnight, sleep-associated, memory consolidation process to favour these events, giving rise to very long-lasting representations.

## 6. An appreciation

Neuroscience is maturing as a discipline and, despite frequent comments to the contrary, we now know a great deal more about the brain than we did in 1971. However, the relationship between its empirical and theoretical branches is far from mature. The modelling philosophy and methodology that Marr created for his three detailed models of the 1970s, and then substantially refined in *Vision* in 1982, remains influential in theoretical circles. However, it is at risk of being forgotten by the wider community where addressing mechanistic issues without thinking about the functions or algorithms performed in a brain area appears prevalent. In an essay broadly sympathetic to the original tri-partite structure, Marr's former colleague Tommy Poggio finds himself wondering how evolution and learning would fit in and whether they also should be considered as separate levels [90]. An additional concern is that the field is now polarizing around either implementational or computational explanations, with the danger of the two never meeting. Marr's legacy bears significantly on the profitability of current endeavours of each type, including hypothesis-free ‘omic’ neuroscience; for example, the collection of immense amounts of data about the connections between all neural elements [91] or global-scale mathematical modelling without a specific computation in mind, such as attempts by the Human Brain Project to build huge-scale simulations of the brain (see https://www.humanbrainproject.eu). The same could be said of implementation-free computational approaches that aim to explore whether the brain functions according to optimality principles.

Within the milieu of theoreticians seeking support for their general theories of the brain, Marr was one of the first to investigate whether specific computational tasks can be implemented on the available neural machinery. A common reading of his later work is that it is appropriate and sufficient to start from the computational level; divorced from implementational considerations, information processing can be readily formulated as optimal inference and control, using ideas from fields such as statistics, operations research, economics and machine learning. However, as recognized throughout the book *Vision*, these accounts are limited. In all but the very simplest circumstances, optimal inference and control are radically intractable for animal or machine alike, and so are formally limited or even useless. It is essential to use heuristics and approximations to the original computational specification. The viability and ultimate performance of a heuristic depends critically on the properties of the substrate on which it is implemented. This opens a critical channel of reverse communication between Marr's three levels. Marr recognizes this point in *Vision* [6, ch. 7], which features imaginary dialogues between a defender of the top-down approach and a sceptic, based on conversations between himself, Tommy Poggio and Francis Crick. In one exchange he accepts that the available neural infrastructure may force a ‘poor man's version’ of the computation to be implemented rather than the computation itself [6, p. 339].

Bottom-up accounts that focus purely on the implementation are attractive because they treat neuroscience as any other natural science. This provides a transparent way to construct models of neural phenomena at multiple scales of investigation. However, these endeavours face two problems. The first is that, as explained at length in *Vision*, such accounts ignore the information processing problems—including the fundamental problem of *representation* that is central to understanding the brain but is irrelevant, or certainly less relevant, in most other domains of natural science. For example, for the case of memory, without the notion of adequate retrieval of past patterns from partial information, the elements and neural circuits of the hippocampus would seem incomprehensibly complicated. As evident in Marr's treatments, such concepts can be a key source of constraints on the structure of the implementation, which is most valuable in the face of the magnitude of the problem. The second problem is one of heterogeneity, which is more subtle and also more pernicious. Conventional approaches to modelling natural phenomena over multiple scales depend critically on homogeneity, i.e. that the innumerable entities at the finer levels of description (such as the sextillions of molecules in a gas, or the million or so cortical nephrons in a human kidney, or the roughly 6000 sodium channels at a node of Ranvier of an axon) can be treated as being at least statistically equivalent. These statistics can then be averaged over time and/or space to derive approximate laws of bulk behaviour applicable at a less detailed temporal or spatial scale (such as the gas laws in physics, or the Hodgkin–Huxley equations; [92]). This approach is the bread-and-butter of statistical physics, with the macroscopic measures reflecting the average properties of microscopic interactions. However, many aspects of the brain are highly heterogeneous over many temporal and spatial scales.

One implementational approach to heterogeneity is just to measure it in all its complex richness—the ‘omics’ strategy. However, the number of such measurements is impossibly large even for a single organism (e.g. the location of every ion channel on every dendrite). Worse, in a strongly nonlinear system such as the brain in which microscopic changes can have macroscopic effects, generalization across time, and between individuals, is very hard. Building a nominally faithful bottom-up simulation, as the Human Brain Project aims to do, is equally problematic.

A second implementational approach is to assume that the heterogeneity of the brain arises from a deeper form of homogeneity, for instance, through a statistical sampling process, and to try to work with the latter. One example is Marr's assumption that the input patterns of activity are generated from independent samples drawn from a simple distribution. Another is Peter's principle that neurons choose to make synapses randomly whenever an axon is sufficiently close to a dendrite [93]. Unfortunately, such simple relationships do not seem to hold—input patterns will actually contain substantial correlations from shared coding; in consequence, cortical wiring exhibits higher order relationships in which generating and verifying more complicated forms is hard [94,95].

An alternative approach to the heterogeneity problem is to argue that it arises through contingency, being tightly regulated to realize algorithmic goals. The heterogeneity will therefore reflect the developmental trajectory of the organism—the explanation for the precise strength of connections between excitatory and inhibitory cells would be merely that it occurs to ensure that excitation does not outweigh inhibition and cause instability. Such effects would only be apparent in deeply buried patterns of correlations in ‘omic’ observations, ones that it would probably be impossible to extract without the algorithmic understanding.

## 7. Conclusion

David Marr sits comfortably with such luminaries as Norbert Wiener, Warren McCulloch, Walter Pitts, Horace Barlow and Donald MacKay as one of the most notable early theoretical neuroscientists. Marr's *Philosophical Transactions* paper appeared in 1971, ironically the year of the first annual meeting of the Society for Neuroscience—a meeting that now attracts 30 000 attendees. Few of them may now know of Marr, though many should. As we have tried to reflect, for a paper that firmly embraces the complexities of the hippocampus (as opposed to concentrating on important but narrower questions such as the mechanism by which spikes are generated), it is quite remarkable how relevant it remains to this day despite so much more now being known. Marr was notably visionary, as well as impressively brave, and he went to great lengths to show how his theories can, at least in principle, be falsifiable. The Royal Society can take credit for recognizing a special talent and allowing him to publish his ideas at length in its journals.

Nevertheless, despite the prescience of this and his other papers, their enduring legacy comes through their influence on contemporary ideas about understanding particular systems of the brain in terms of the computations they carry out with the neural hardware available. His methodology gave the intellectual infrastructure within which almost all subsequent modelling has been performed. He thereby provided a means to establish the communication between levels that is necessary to make lasting progress, a lesson that modern attempts that focus too narrowly on one level at the expense of others ignore at their peril.

## Author profiles

**David Willshaw** is Emeritus Professor of Computational Neurobiology at the University of Edinburgh. Prior to this, he was a member of MRC External Scientific Staff and then University of Edinburgh staff, with Programme Grant support from both MRC and The Wellcome Trust. His main research interest is in modelling the development of nerve connections in both visual and neuromuscular systems. His earlier research was on models of learning and memory, as represented by his PhD topic on modelling distributed associative memory, contemporary with David Marr's study on the archicortex. Together with students, he has used computer simulation studies to explore the properties of Marr's models of cerebellar cortex, neocortex and archicortex.

**Peter Dayan** is Director of the Gatsby Computational Neuroscience Unit at UCL. He did his PhD with David Willshaw in the University of Edinburgh, and completed postdocs with Terry Sejnowski at the Salk Institute and Geoff Hinton at the University of Toronto. He was appointed an assistant professor at MIT, and then moved to London in 1998. His interests span decision-making, representational and reinforcement learning, and associative memory.

**Richard Morris** is Professor of Neuroscience at the University of Edinburgh. He completed a DPhil at the University of Sussex and was first appointed as a lecturer in St Andrews prior to moving to Edinburgh in 1986. His main research interests are in the neurobiology of learning and memory, with a particular focus on hippocampal function. He was instrumental in creating the interdisciplinary organization ‘Edinburgh Neuroscience’ which endeavours to bring together scientists interested in the brain using a variety of distinct approaches, encompassing both basic and clinical approaches.
